# A10 PROLINE METABOLISM AFFECTS CANCER STEM CELLS IN ESOPHAGEAL SQUAMOUS CELL CARCINOMA

**DOI:** 10.1093/jcag/gwad061.010

**Published:** 2024-02-14

**Authors:** A Igouzoul, J Douchin, E Audet-Walsh, F Boisvert, V Giroux

**Affiliations:** Universite de Sherbrooke Faculte de Medecine et des Sciences de la Sante, Sherbrooke, QC, Canada; Universite de Sherbrooke Faculte de Medecine et des Sciences de la Sante, Sherbrooke, QC, Canada; Universite Laval Faculte de Medecine, Quebec, QC, Canada; Universite de Sherbrooke Faculte de Medecine et des Sciences de la Sante, Sherbrooke, QC, Canada; Universite de Sherbrooke Faculte de Medecine et des Sciences de la Sante, Sherbrooke, QC, Canada

## Abstract

**Background:**

Esophageal squamous cell carcinoma (ESCC) is highly deadly with a 5-year survival rate of only 16%, partly due to treatment resistance. Resistance is associated, among others, with the presence of cancer stem cells (CSC). Previous work in the laboratory has shown that prolonged exposure to anticancer treatments such as radiation and/or 5-FU leads to increased number of CSCs. Interestingly, disruption of amino acid metabolism, especially decreased proline levels, has been observed in treated cells. Proline is a non-essential amino acid that can either be uptake from the environment or synthesized from glutamate or ornithine. However, its role in cancer cells remains poorly understood and controversial.

**Aims:**

Therefore, my project aims at determining the role of proline metabolism in CSC abundance and tumoral properties in ESCC.

**Methods:**

Using ESCC cell lines, TE11 and TE15, we stimulated proline metabolism by adding proline to the media or inhibited its catabolism using L-THFA. L-THFA forces proline accumulation in the cell. We then conducted flow cytometry to quantify CSC proportion, proliferation assay, spheroid formation assay and spheroids included in collagen for migration and invasion assay.

**Results:**

First, the stimulation of proline metabolism decreased the proportion of CSCs in flow cytometry. These results align with our initial observations, where CSC-enriched cells exhibited reduced proline levels. Second, the inhibition of proline catabolism by L-THFA had the opposite effect, namely an increase in the proportion of CSC. Interestingly, these changes in the proportion of CSCs induced by proline or L-THFA translated into changes in clonogenic potential, a capacity typically associated with stem cells. Indeed, limiting dilution spheroids formation assays showed that proline decreased while L-THFA increased the number of formed spheroids. Since spheroids formation is linked to the presence of CSC, this provides new evidence of the impact of proline metabolism on CSC characteristics. Finally, L-THFA treatments drastically increased migratory and invasive abilities in 3D spheroid models compared to untreated or proline-treated conditions.

**Conclusions:**

In conclusion, our results suggest that proline metabolism is implicated in the enrichment of cancer stem cells as well as in their functions.

**Funding Agencies:**

CAG, CIHRinnovation.ca ; Centre de recherche CHUS ; FMSS ; Canada Research Chairs

